# Hints to the People upon the Profession of Medicine

**Published:** 1852-02

**Authors:** 


Art. VIII.—*Hints to the People upon the Profession of Medicine.* By William Maxwell Wood, M. D., Surgeon U. S. Navy: author of "Sketches of South America," "Polynesia," etc. Buffalo: Geo. H. Derby & Co. 1852.

This neat little volume is a reprint from the Buffalo Medical Jourual, and does its author a great deal of credit. There is a vast amount of useful information for the people, contained in this monograph, and students of medicine may glean from its pages, many valuable lessons, which, if properly used, may throw light in advance of their footsteps. Coleridge says that "the experience of most men is like the stem lights of a ship, which only lights the track already passed over," but if Dr. Wood's light are discreetly used, they may throw light ahead.

We cordially thank Dr. Wood for this well conceived, and well executed brochure. It is an honor to him, and if read by the proper parties, will do much good to the people, and to medical men. We understand that the worthy publishers are selling the pamphlet in quantities at a small price, in order to secure an extensive dissemination of its valuable truths. If it were a political pamphlet, as well calculated to advance the interests of a party, as this is to improve the position of the medical profession, orders would flow in upon the publishers. Shall medical men do less for their nobie profession, than political partisans do for the evanescent glories of their creeds?

